# Association of vitamin D deficiency and subclinical diabetic peripheral neuropathy in type 2 diabetes patients

**DOI:** 10.3389/fendo.2024.1354511

**Published:** 2024-03-25

**Authors:** Xiaoyang Sun, Xinyu Yang, Xiaopeng Zhu, Yu Ma, Xu Li, Yuying Zhang, Qiling Liu, Chenmin Fan, Miao Zhang, Binger Xu, Yanlan Xu, Xin Gao, Jihong Dong, Mingfeng Xia, Hua Bian

**Affiliations:** ^1^Department of Endocrinology, Zhongshan Hospital, Fudan University, Shanghai, China; ^2^Institute of Metabolic Disease, Fudan University, Shanghai, China; ^3^Department of Neurology, Zhongshan Hospital, Fudan University, Shanghai, China; ^4^Institute of Metabolism &Integrative Biology (IMIB), Fudan University, Shanghai, China; ^5^Department of Geriatrics, Qingpu Branch of Zhongshan Hospital, Fudan University, Shanghai, China

**Keywords:** vitamin D, diabetic peripheral neuropathy, distal symmetric polyneuropathy, type 2 diabetes, nerve conduction study (NCS)

## Abstract

**Background:**

Diabetic peripheral neuropathy (DPN) contributes to disability and imposes heavy burdens, while subclinical DPN is lack of attention so far. We aimed to investigate the relationship between vitamin D and distinct subtypes of subclinical DPN in type 2 diabetes (T2DM) patients.

**Methods:**

This cross-sectional study included 3629 T2DM inpatients who undertook nerve conduction study to detect subclinical DPN in Zhongshan Hospital between March 2012 and December 2019. Vitamin D deficiency was defined as serum 25-hydroxyvitamin D (25(OH)D) level < 50 nmol/L.

**Results:**

1620 (44.6%) patients had subclinical DPN and they were further divided into subgroups: distal symmetric polyneuropathy (DSPN) (n=685), mononeuropathy (n=679) and radiculopathy (n=256). Compared with non-DPN, DPN group had significantly lower level of 25(OH)D (P < 0.05). In DPN subtypes, only DSPN patients had significantly lower levels of 25(OH)D (36.18 ± 19.47 vs. 41.03 ± 18.47 nmol/L, P < 0.001) and higher proportion of vitamin D deficiency (78.54% vs. 72.18%, P < 0.001) than non-DPN. Vitamin D deficiency was associated with the increased prevalence of subclinical DPN [odds ratio (OR) 1.276, 95% confidence interval (CI) 1.086-1.501, P = 0.003] and DSPN [OR 1. 646, 95% CI 1.31-2.078, P < 0.001], independent of sex, age, weight, blood pressure, glycosylated hemoglobin, T2DM duration, calcium, phosphorus, parathyroid hormone, lipids and renal function. The association between vitamin D deficiency and mononeuropathy or radiculopathy was not statistically significant. A negative linear association was observed between 25(OH)D and subclinical DSPN. Vitamin D deficiency maintained its significant association with subclinical DSPN in all age groups.

**Conclusions:**

Vitamin D deficiency was independently associated with subclinical DSPN, rather than other DPN subtypes.

## Highlights

- The relationship between vitamin D and distinct subtypes of subclinical DPN remains unclear.
- We detected 25(OH)D level and nerve conduction study in 3629 T2DM inpatients.
- Subclinical DSPN patients had significantly lower levels of 25(OH)D and higher proportion of vitamin D deficiency.
- Vitamin D deficiency was independently associated with the increased prevalence of subclinical DSPN in all age groups, rather than mononeuropathy or radiculopathy.

## Introduction

Diabetic peripheral neuropathy (DPN) ranks among the most prevalent complications of diabetes and is present in more than 50% of patients with type 2 diabetes (T2DM) aged over 60 years (1). DPN encompasses conditions such as distal symmetric polyneuropathy (DSPN), radiculopathy, mononeuropathy, as well as autonomic neuropathy or treatment-induced neuropathy (2). DPN significantly contributes to disability in diabetes, affecting patients’ overall quality of life and imposing substantial economic burdens on society (3, 4). The early prevention and management of DPN is of great importance. However, over half of DPN patients are asymptomatic (5). Timely and intensive interventions can improve subclinical DPN and reduce the risk of DPN progression (5–7).

Vitamin D deficiency has emerged as a progressively serious global concern, with association with multiple diseases such as cardiovascular diseases, metabolic syndrome, cancer, autoimmune disorders, and Alzheimer’s disease (8). Beyond its role in regulating calcium, phosphorus, and bone metabolism, vitamin D is also implicated in insulin secretion and resistance (9). Vitamin D insufficiency is prevalent among T2DM patients (10, 11). Recently, the correlation between vitamin D and DPN has garnered increasing attention. While existing cross-sectional studies have indicated relationship between vitamin D deficiency and the risk of DPN (12–16), these studies often have limitations such as small sample sizes (15) or reliance on subjective diagnostic criteria (12). Previous research relied on clinical symptoms and/or physical signs, providing limited evidence regarding the association between vitamin D and subclinical DPN. Furthermore, the precise relationship between vitamin D and distinct subtypes of DPN remains unclear at present.

This study aims to clarify the relationship between vitamin D and subclinical DPN, as well as its distinct subtypes including DSPN, radiculopathy and mononeuropathy by recruiting a large sample of asymptomatic DPN individuals verified through nerve conduction study (NCS). We seek to provide evidence that contributes to the early prevention and treatment of DPN.

## Methods

### Study population

Between March 2012 and December 2019, T2DM patients from the endocrinology ward of Zhongshan Hospital, Fudan University, were enrolled as participants. Eligibility criteria included age ranging from 18 to 70 years, a diagnosis of T2DM (17), and no neurological symptoms but undergoing nerve conduction study. Among the criteria for exclusion were Type 1 diabetes or other special types of diabetes, with other causes of peripheral neuropathy, such as cervical and lumbar spine disorders, autoimmune diseases, as well as nerve damage attributed to medication or alcohol abuse. Written informed consents were obtained from all patients. The study was approved by the Ethics Committee of Zhongshan Hospital Fudan University and adhered to the tenets of the Declaration of Helsinki.

### Neuropathy assessment

All participants underwent electromyography and nerve conduction study. Needle electromyography was performed to exclude the participants with potential muscle diseases in a quiet environment as described in a previous study (18). Electrical activities of the tested muscle were measured in both resting and different contraction states after inserting a concentric circular needle electrode. NCS was performed using a Dandy Keypoint electromyography instrument. A unilateral nerve conduction examination of the upper (median and ulnar motor and sensory nerves) and lower limb nerves (peroneal and tibial motor nerves and the sural sensory nerve) was conducted while maintaining the skin temperature at about 34°C. Conduction velocities, amplitudes of sensory and motor nerves as well as the distal latency and late response of motor nerves were recorded. All results were interpreted by an experienced neurology professor blind to the clinical information.

Subclinical DPN was diagnosed as having at least one abnormal NCS parameter using age- and height-adjusted thresholds for abnormality but did not display any sensory symptoms, signs, or reflex abnormalities. DPN was further classified into different subtypes based on different patterns of electrophysiological abnormalities. DSPN manifests as symmetrical nerve conduction velocity or amplitude abnormalities in the lower (and upper) extremities. Mononeuropathy manifests as asymmetric distal nerve conduction abnormalities. Radiculopathy manifests with asymmetrical proximal nerve conduction abnormalities in the absence of myogenic damage on electromyography. Autonomic neuropathy was not included in the scope of this research. On the other hand, the non-DPN group was composed of patients who did not present with clinically evident DPN or display normal results in nerve conduction tests.

### Clinical data collection

Data for age (y), sex, duration of diabetes (y), height (m), and weight (kg) were collected. The body mass index (BMI) was calculated using the formula BMI (kg/m²) = (weight in kg)/(height in meters)². Blood samples were collected from the antecubital vein to assess total cholesterol (TC), triglyceride (TG), high-density lipoprotein cholesterol (HDL-C), low-density lipoprotein cholesterol (LDL-C), serum creatinine (sCr), and uric acid (UA). These measurements were conducted using an enzymatic method with an automated biochemical analyzer (7600-020, Hitachi Inc., Tokyo, Japan). Glycosylated hemoglobin (HbA1c) levels were determined using high-pressure liquid chromatography on the Variant™ II machine (Bio-Rad, Hercules, CA, USA). The estimated glomerular filtration rate (eGFR) was calculated using the CKD-EPI Creatinine Equation (19). Parathyroid hormone levels were assessed through an electrochemiluminescence assay (Roche, Mannheim, Germany). The value of vitamin D was obtained using an electrochemiluminescence assay (Roche) for 25-dihydroxyvitamin D [25(OH)D] serum concentration. Vitamin D deficiency (VDD) was defined as serum circulating 25(OH)D level < 50 nmol/L (20 ng/mL) (20).

### Statistical analysis

The data were analyzed using R version 4.0.3. For normal distributed continuous variables, data were expressed as mean ± standard deviation. To compare two groups, an independent samples t-test was applied. For non-normal distributed continuous variables, data were presented as median (quartile). When comparing two groups, the Mann-Whitney U test was utilized. Categorical variables were represented as percentages (%). The chi-square test or Fisher’s exact test was employed to compare these categorical variables. Multiple logistic regression analysis was performed to evaluate the odds ratio (OR) and associated factors. The OR (95% confidence interval) for DPN in relation to 25(OH)D was calculated across three logistic regression models: a non-adjusted model, an age and sex-adjusted model, and a multivariable model adjusted for various variables, including sex, age, BMI, blood pressure, HbA1c, T2DM duration, eGFR, calcium, phosphorus, PTH, TC, TG, LDL-C, and HDL-C. ORs and 95%CIs of subclinical DSPN and vitamin D deficiency across different age were estimated in a similar way, and their interaction was tested. Restricted cubic splines (RCS) with four knots at the 5th, 35th, 65th, and 95th centiles were employed to flexibly model and describe relationships between the 25(OH)D and the DSPN. All P values were two-tailed, and P < 0.05 was considered as statistically significant.

## Results

### Comparison of variables between non-DPN and subclinical DPN

**Figure 1:**
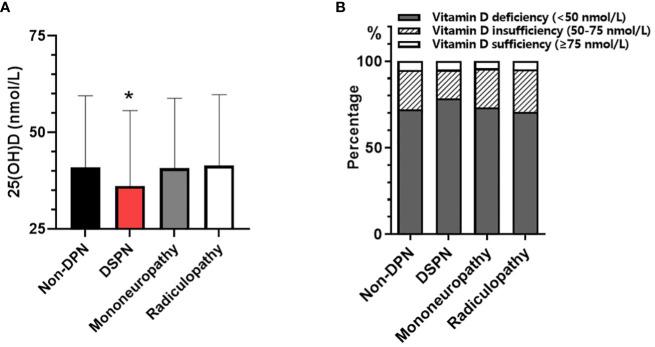
Comparison of serum 25(OH)D concentration among patients with non-DPN and different types of subclinical DPN: **(A)** serum 25(OH)D level; **(B)** prevalence of vitamin D deficiency. *p<0.05, vs. Non-DPN.

The study population (n = 3629, 2155 men and 1474 women) had a mean age of 57.73 ± 13.22 years, and had been diagnosed with T2DM for 7(1~12) years. Overall, compared with non-DPN group, patients with subclinical DPN had older age, a longer duration of diabetes, poorer glycemic control, and impaired kidney function. Compared with non-DPN, DPN group had significantly lower 25(OH)D level (38.94 ± 18.87 vs. 41.03 ± 18.47 nmol/L, P < 0.05). In DPN subtypes, only patients in DSPN subgroup had significantly lower levels of 25(OH)D (36.18 ± 19.47 vs. 41.03 ± 18.47 ng/mL, P < 0.001) and higher proportion of vitamin D deficiency (78.54% vs. 72.18%, P<0.001) than non-DPN group (**Figure 1**). However, these differences did not display significant in patients with mononeuropathy or radiculopathy (**Table 1**).

**Table 1:** Characteristics of diabetes patients without neurological symptoms who underwent electromyogram.

	Non-DPN (N=2009)	Subclinical DPN (N=1620)			
		All (N=1620)	Distal symmetric polyneuropathy (N=685)	Mononeuropathy (N=679)	Radiculopathy (N=256)
Male [n(%)]	1229 (61.17%)	926 (57.16%)*	497 (72.55%)ΔΔ	270 (39.76%)##	159 (62.11%)
Age (y)	55.22 ± 13.49	60.83 ± 12.2**	59.66 ± 14.16ΔΔ	61.57 ± 10.21##	61.99 ± 11.11↑↑
Duration of T2DM (y)	5 (0.9-10)	10 (3-15)**	10 (5-16)ΔΔ	8 (2-15)##	7 (1.38-13)↑
Weight (kg)	70.26 ± 13.45	69.78 ± 13.13	70.09 ± 13.06	69.14 ± 12.85#	70.64 ± 14.02
Body mass index (kg/m^2^)	25.6 ± 3.86	25.79 ± 3.94	25.1 ± 3.84Δ	26.5 ± 3.92##	25.73 ± 3.92
Systolic blood pressure (mmHg)	131.51 ± 17.19	133.84 ± 17.78**	133.17 ± 18.77Δ	134.44 ± 17.17##	134.02 ± 16.6↑
Diastolic blood pressure (mmHg)	79.53 ± 10.15	78.64 ± 10.31*	78.33 ± 10.54Δ	78.21 ± 10.17#	80.59 ± 9.86
HbA1c (%)	8.77 ± 2.16	9.26 ± 2.19**	9.56 ± 2.28ΔΔ	8.99 ± 1.98#	9.17 ± 2.38↑
Total cholesterol (mmol/L)	4.36 (3.74-5.05)	4.26 (3.57-5.08)*	4.2 (3.54-5)Δ	4.29 (3.62-5.13)	4.26 (3.56-5.1)
Triglycerides (mmol/L)	1.49 (1.04-2.27)	1.38 (0.98-2.08)**	1.33 (0.92-2.02)ΔΔ	1.44 (1.01-2.14)	1.33 (1-2.05)↑
LDL cholesterol (mmol/L)	2.44 (1.91-3.05)	2.42 (1.81-3.07)	2.36 (1.82-3.07)	2.47 (1.83-3.09)	2.41 (1.7-3.02)
HDL cholesterol (mmol/L)	1.04 (0.88-1.27)	1.08 (0.9-1.31)*	1.06 (0.88-1.32)	1.08 (0.92-1.31)#	1.08 (0.9-1.3)
Serum creatinine (μmol/L)	67 (56-79)	67 (56-83)*	70 (59-92)ΔΔ	63 (54-77)##	68 (57-81)
Uric acid (μmol/L)	310 (252-376)	303 (250-371)	318 (254.5-389.5)	290 (245.5-362)#	303(251-363)
eGFR (mL/min/1.73m^2^)	92.12 (73.82-103.67)	88.64 (66.93-101.85)**	83.67 (59.91-99.88)ΔΔ	93.95 (75.7-103.59)	87.85(69.53-100.65)↑
Calcium (mmol/L)	2.27 ± 0.12	2.25 ± 0.13	2.24 ± 0.14ΔΔ	2.27 ± 0.11#	2.25 ± 0.13
Phosphate (mmol/L)	1.24 ± 0.2	1.22 ± 0.21*	1.2 ± 0.24ΔΔ	1.24 ± 0.19	1.22 ± 0.2
Parathyroid hormone (ng/L)	37 (29.1-47.1)	35.75 (26.8-46.62)**	33.9 (25.4-46.6)ΔΔ	36.7 (28.15-46.95)	36.05 (27.3-45.65)
25(OH)D (nmol/L)	41.03 ± 18.47	38.94 ± 18.87*	36.18 ± 19.47ΔΔ	40.78 ± 18.09	41.41 ± 18.41
Vitamin D deficiency [n(%)]	1450 (72.18%)	1216 (75.06%)	538 (78.54%) ΔΔ	497 (73.20%)	181 (70.70%)

DPN, diabetic peripheral neuropathy; T2DM, type 2 diabetes; HbA1c, glycosylated hemoglobin; LDL, low-density lipoprotein; HDL, high-density lipoprotein; eGFR, estimated glomerular filtration rate.

*P<0.05, **P<0.001, vs. non-DPN.

ΔP<0.05, ΔΔP<0.001, vs. non-DPN.

#P<0.05, ##P<0.001, vs. non-DPN.

↑P<0.05, ↑↑P<0.001, vs. non-DPN.

### Comparison of characteristics between groups with different levels of 25(OH)D

The diabetic patients were categorized into two groups based on their 25(OH)D levels: less than 50 nmol/L (20 ng/mL) (vitamin D deficiency), and greater than or equal to 50 nmol/L. In the vitamin D deficiency group, a lower proportion of males, younger age, higher BMI, worse control of glucose and lipids, lower blood calcium levels, and higher PTH levels were observed. Notably, the proportion of subclinical DSPN significantly increased in patients with vitamin D deficiency (20.18% vs. 15.26%, P=0.001), while no similar trend was found in mononeuropathy or radiculopathy (**Supplementary Figure S1**, **Supplementary Table S1**).

### Association of vitamin D deficiency with different subtypes of subclinical DPN

To further investigate the relationship between vitamin D deficiency and different subtypes of subclinical DPN, a multiple logistic regression analysis was conducted with and without subclinical DPN as the dependent variable. Even after adjusting for age and sex, a significant association between subclinical DPN and vitamin D deficiency remained (OR 1.237, 95% CI 1.062-1.442, P = 0.006). In the multivariable model that adjusted sex, age, weight, blood pressure, HBA1C, T2DM duration, calcium, phosphorus, parathyroid hormone, lipids and renal function, vitamin D deficiency maintained its significant association with subclinical DPN (OR 1.276, 95% CI 1.086-1.501, P = 0.003). Similarly, in the case of the DSPN subtype, vitamin D deficiency was related with increased risk of subclinical DSPN (OR 1.646, 95% CI 1.31-2.078, P < 0.001) after adjusting for multiple variables. However, the association between vitamin D deficiency and subclinical mononeuropathy or radiculopathy was not statistically significant (**Table 2**). These findings highlight a robust correlation between vitamin D deficiency and the subclinical DSPN, with less substantial evidence for its link to other subtypes of subclinical DPN.

**Table 2:** Odds ratios of vitamin D deficiency contributing to different types of subclinical DPN.

	N		OR (95%CI)	P value
DPN	1620	Non-adjusted	1.16 (1-1.347)	0.05
		Age-and sex-adjusted	1.237 (1.062-1.442)	0.006
		Multivariate adjusted	1.276 (1.086-1.501)	0.003
DSPN	685	Non-adjusted	1.411 (1.15-1.739)	0.001
		Age-and sex-adjusted	1.584 (1.285-1.962)	<0.001
		Multivariate adjusted	1.646 (1.31-2.078)	<0.001
Mononeuropathy	679	Non-adjusted	1.053 (0.867-1.283)	0.607
		Age-and sex-adjusted	1.055 (0.862-1.295)	0.606
		Multivariate adjusted	1.101 (0.889-1.368)	0.38
Radiculopathy	256	Non-adjusted	0.93 (0.702-1.245)	0.621
		Age-and sex-adjusted	1.017 (0.763-1.368)	0.91
		Multivariate adjusted	1.012 (0.747-1.381)	0.941

Multivariate adjusted: adjusted for sex, age, body mass index, blood pressure, glycosylated hemoglobin, T2DM duration, estimated glomerular filtration rate, calcium, phosphorus, parathyroid hormone, total cholesterol, triglyceride, low-density lipoprotein cholesterol and high-density lipoprotein cholesterol.

DPN, diabetic peripheral neuropathy; DSPN, Distal symmetric polyneuropathy; T2DM, type 2 diabetes.

**Figure 2:**
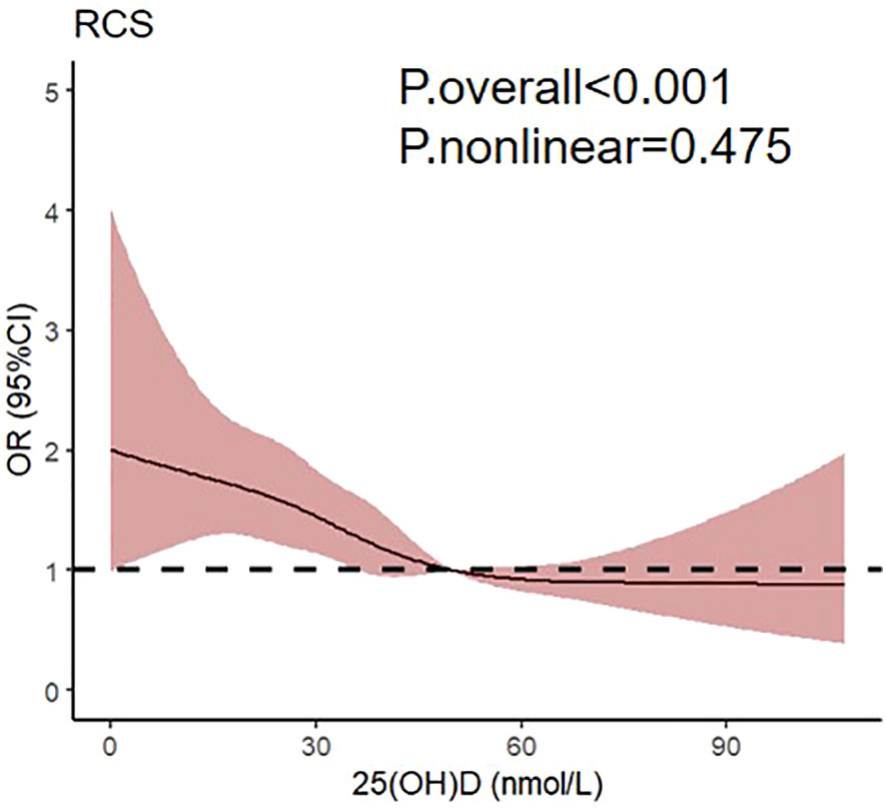
Relationship between 25(OH)D and subclinical DSPN using restricted cubic splines.

To further investigate the correlation between 25(OH)D and subclinical DSPN, the restricted cubic spline regression with multivariate adjusted model was visualized (**Figure 2**). Notably, a linear association was observed between 25(OH)D levels and subclinical DSPN in patients with type 2 diabetes, with the odds ratio of subclinical DSPN exhibiting a significant increase as 25(OH)D levels decreased below 50 nmol/L.

### Association of vitamin D deficiency with subclinical DSPN across different age

To further explore the impact of age on the correlation between vitamin D and subclinical DSPN, we categorized patients according to age after excluding those with other subtypes of DPN: 18 to 44 years (Youth Group, n=499), 45 to 59 years (Middle-Age Group, n=986), and ≥ 60 years (Elderly Group, n=1209). For each age group, patients with subclinical DSPN had significantly lower levels of vitamin D than non-DSPN. The prevalence of vitamin D deficiency was significantly higher in subclinical DSPN for youth (88% vs. 77.94%, P = 0.035) and elderly subgroup (76.02% vs. 69.89%, P = 0.031), and the difference was near to statistical significance in middle-age group (78.76% vs. 71.63%, P=0.056) (**Supplementary Table S2**). After multivariate adjustment, vitamin D deficiency maintained its significant association with subclinical DSPN in youth (OR 2.427, 95% CI 1.201-5.308, P = 0.018), middle-age group (OR 1.96, 95% CI 1.288-3.039, P = 0.002) and elderly group (OR 1.439, 95% CI 1.061-1.961, P = 0.02) (**Supplementary Table S3**).

## Discussion

In this study, we aimed to investigated the association between vitamin D and subclinical diabetic peripheral neuropathy in T2DM patients. Our results show that in all age groups, vitamin D deficiency was related to a higher risk of DSPN and this relationship was independent of sex, age, weight, blood pressure, HbA1c, T2DM duration, calcium, phosphorus, parathyroid hormone, lipids and renal function. However, we didn’t find a similar relationship between vitamin D and other subtypes of DPN, like mononeuropathy or radiculopathy. These findings suggest new possibilities for better DPN screening and treatment.

The association between vitamin D and DPN has been reported. Significantly lower vitamin D levels were found in DPN patients (36.9 nmol/L), compared to non-DPN patients (58.32 nmol/L) (15). In a 25-year observational study, individuals with 25(OH)D < 50 nmol/L experienced a higher cumulative incidence of macrovascular and microvascular events than those with 25(OH)D ≥ 50 nmol/L (21). Similarly, cross-sectional studies have indicated that insufficient vitamin D levels might increase the risk of DPN. In a study including 861 T2DM patients, multivariate logistic regression analysis showed that vitamin D deficiency was an independent factor contributing to DPN (β=0.88, P<0.01) (16). The severity of vitamin D deficiency was also closely correlated with the severity of neuropathy (22, 23). Clinical trials involving topical compounds containing vitamin D (24) or vitamin D supplementation have demonstrated significant therapeutic benefits in DPN patients (22). Similarly, our study confirmed the inverse correlation between vitamin D and DPN, specifically within the DSPN subtype. A previous study utilizing restricted cubic splines analysis proposed a nonlinear connection between vitamin D and DPN, where the risk of DPN remained notable until vitamin D reached 30 nmol/L, after which it started to lose significance. In essence, lower vitamin D levels might increase the risk of DPN (25). In our outcomes, a linear relationship emerged between vitamin D and subclinical DSPN, with the odds ratio of subclinical DSPN exhibiting a significant increase as 25(OH)D levels decreased below 50 nmol/L. Our cohort comprised individuals with subclinical DPN devoid of neurological symptoms, which diverged from previous typical DPN studies, potentially accounting for results inconsistency. Our findings suggested that vitamin D supplementation may potentially delay the occurrence of subclinical DSPN, which held considerable implications for the early prevention and management of DSPN.

Older adults are at risk for vitamin D deficiency as a result of decreased dietary intake and calcium absorption, as well as decline in renal function (26). Yang Niu (27) reported that low vitamin D was associated with DPN in patients of ≥ 65 years but not in young and middle-aged patients. However, our study found independent association between vitamin D deficiency and subclinical DSPN in all age groups. The cause for the difference may be due to the different cohort (a larger sample size and more young patients) and different diagnostic criteria (subclinical or asymptomatic DSPN). The result highlights the importance of preventing vitamin D deficiency in non-elderly people.

The potential mechanisms of vitamin D improving DSPN may include the following aspects: 1) Neuroprotection: Vitamin D could potentially shield neuronal cells against apoptosis and neurodegeneration. Vitamin D could trigger the generation of nerve growth factors (28–32), contributing to the preservation of neurons. In diabetic rats, an elevated expression of vitamin D receptors in neurons of the dorsal root ganglion bolsters their sensitivity (33). 2) Enhanced blood circulation: Vitamin D deficiency in rodents lead to compromised vascular relaxation and capillary constriction, diminished antioxidant activity, and heightened endothelium-dependent contraction (34, 35). Thus vitamin D might play a role in maintaining proper blood circulation by facilitating angiogenesis and endothelial repair. 3) Anti-inflammatory effects: Clinical research has suggested that administering high doses of vitamin D to patients with DPN was linked to a reduction in serum IL-6 levels and an elevation in IL-10 concentrations, implying alleviated inflammation (36). DSPN is frequently related to a chronic course and stems from fiber-predominant small nerve lesions, primarily induced by factors such as glucotoxicity, lipotoxicity, oxidative stress, impaired endothelial cell function, dysregulation of the Na+-K+-ATP pump, and endoplasmic reticulum stress (37). These mechanisms are related to neurotrophic disorders. In contrast, radiculopathy often has acute onset, encompassing the lumbar-sacral or cervical plexus and is associated with ischemic damage and microvascular inflammation. Mononeuropathy typically affects oculomotor and median nerves, and may be related to compression or ischemia. Vitamin D mainly plays a role in promoting the production of nerve growth factor and improving microcirculation, which may account for its stronger correlation with chronic progression of DSPN in our study. However, whether the mechanisms underlying non-DSPN neuropathies share similarities remains uncertain (38). Distinct mechanisms underlying the development of various DPN subtypes also require further investigation.

This study has several strengths. Firstly, we employed nerve conduction study to accurately identify subclinical DPN in asymptomatic T2DM patients, enabling a focused analysis of risk factors specific to subclinical DPN. Secondly, our study included different subtypes of subclinical DPN and featured a relatively substantial sample size. Additionally, our research introduced a novel finding: Vitamin D deficiency was associated with the increased prevalence of subclinical DSPN rather than other DPN subtypes. And there was a linear association between 25(OH)D and subclinical DSPN.

Nevertheless, the current study has some limitations. Firstly, as it was a cross-sectional study, the long-term outcomes for patients with different 25(OH)D levels remain unknown. Secondly, vitamin D levels are affected by factors like ethnicity, geographical location, dietary habits, sunlight exposure, and variations in single nucleotide polymorphisms of vitamin D receptor. Thirdly, the quantitative analysis of the correlation between 25(OH)D levels and neuro-conduction parameters was not conducted in this study.

In summary, it is advisable to enhance the screening and assessment of DSPN in diabetes patients with vitamin D deficiency. Avoid vitamin D deficiency could potentially offer advantages in preventing subclinical DSPN. Nonetheless, more large-scale, forward-looking, and multi-center investigations are needed to unravel the role of vitamin D in preventing and treating DSPN.

## Conclusion

In conclusion, our study demonstrated that vitamin D deficiency was independently associated with subclinical DSPN in all age groups, rather than other subtypes of DPN. Although further studies are needed to clarify the effect of vitamin D on DSPN development, the study supports that special attention should be paid to the patients with vitamin D deficiency for their susceptibility to DSPN.

## Data availability statement

The original contributions presented in the study are included in the article/**Supplementary Material**. Further inquiries can be directed to the corresponding authors.

## Ethics statement

The studies involving humans were approved by Ethics Committee of Zhongshan Hospital Fudan University [B2021-160(2)]. The studies were conducted in accordance with the local legislation and institutional requirements. The participants provided their written informed consent to participate in this study.

## Author contributions

XS: Visualization, Methodology, Investigation, Formal analysis, Writing – review & editing, Writing – original draft. XY: Data curation, Writing – review & editing, Writing – original draft, Methodology, Investigation. XZ: Writing – review & editing, Writing – original draft, Methodology, Investigation. YM: Writing – review & editing, Writing – original draft, Resources, Project administration, Methodology. XL: Writing – original draft, Formal analysis, Data curation. YZ: Writing – original draft, Investigation. QL: Writing – original draft, Methodology. CF: Writing – original draft, Investigation. MZ: Writing – original draft, Investigation. BX: Writing – original draft, Data curation. YX: Writing – original draft, Validation, Methodology. XG: Writing – review & editing, Conceptualization. JD: Writing – review & editing, Supervision, Methodology, Investigation, Data curation, Conceptualization. MX: Writing – review & editing, Supervision, Investigation, Funding acquisition, Conceptualization. HB: Writing – review & editing, Writing – original draft, Supervision, Project administration, Investigation, Funding acquisition, Data curation, Conceptualization.
