# Supplementary material for: Association of vitamin D deficiency and subclinical diabetic peripheral neuropathy in type 2 diabetes patients
Source: Front Endocrinol (Lausanne). 2024 Mar 25;15:1354511. doi: 10.3389/fendo.2024.1354511 (PMC10999604; doi:10.3389/fendo.2024.1354511)
Supplement: Supplementary file 1 [file DataSheet_1.docx]

Table S1. Comparison of the characteristics between subgroups based on the 25(OH)D level.

|  | 25(OH)D < 50 nmol/L  (Vitamin D Deficiency) | 25(OH)D ≥ 50 nmol/L | P value |
| --- | --- | --- | --- |
|  | (N=2666) | (N=963) |  |
| Male [n(%)] | 1530(57.39%) | 625(64.9%) | <0.001 |
| Age (y) | 57.23±13.62 | 59.09±11.96 | 0.002 |
| Duration of T2DM (y) | 7(1-12) | 8(2-14) | <0.001 |
| Weight (kg) | 70.29±13.61 | 69.35±12.43 | 0.16 |
| Body mass index (kg/m^2^) | 25.81±4 | 25.32±3.58 | 0.001 |
| Systolic blood pressure (mmHg) | 132.5±17.51 | 132.68±17.45 | 0.636 |
| Diastolic blood pressure (mmHg) | 79.23±10.3 | 78.85±10.03 | 0.578 |
| HbA1c (%) | 9.1±2.17 | 8.67±2.19 | <0.001 |
| Total cholesterol (mmol/L) | 4.36(3.69-5.13) | 4.15(3.57-4.85) | <0.001 |
| Triglycerides (mmol/L) | 1.48(1.02-2.26) | 1.33(0.98-1.98) | <0.001 |
| LDL cholesterol (mmol/L) | 2.48(1.87-3.11) | 2.34(1.84-2.91) | 0.001 |
| HDL cholesterol (mmol/L) | 1.05(0.88-1.29) | 1.07(0.9-1.3) | 0.141 |
| Serum creatinine (μmol/L) | 66(55-80) | 69(59-82) | <0.001 |
| Uric acid (μmol/L) | 306(247.5-373) | 313(257-376) | 0.125 |
| eGFR (mL/min/1.73m^2^) | 92.16(71.99-103.95) | 86.7(69.62-99.66) | <0.001 |
| Calcium (mmol/L) | 2.26±0.12 | 2.27±0.12 | <0.001 |
| Phosphate (mmol/L) | 1.24±0.21 | 1.22±0.2 | 0.006 |
| Parathyroid hormone (ng/L) | 37.6(29.13-48.2) | 33.5(26.05-42.3) | <0.001 |
| 25(OH)D (nmol/L) | 31.29±11.01 | 64.45±13.08 | <0.001 |
| Subclinical DPN [n(%)] | 1216(45.61%) | 404(41.95%) | 0.055 |
| DSPN [n(%)] | 538(20.18%) | 147(15.26%) | 0.001 |
| Mononeuropathy [n(%)] | 497(18.64%) | 182(18.9%) | 0.899 |
| Radiculopathy [n(%)] | 181(6.79%) | 75(7.79%) | 0.335 |

Abbrevation: DPN: diabetic peripheral neuropathy, DSPN: distal symmetric polyneuropathy; T2DM: type 2 diabetes, HbA1c: glycosylated hemoglobin, LDL: low-density lipoprotein, HDL: high-density lipoprotein, eGFR: estimated glomerular filtration rate.

Figure S1. Prevalence of subclinical DSPN in subgroups based on the 25(OH)D level.

Table S2. Comparison of the characteristics for patients in three age groups with and without subclinical DSPN.

|  | Youth Group (n=499) | |  | Middle-Age Group (n=986) | |  | Elderly Group (n=1209) | |
| --- | --- | --- | --- | --- | --- | --- | --- | --- |
|  | Non-DSPN (N=399) | DSPN (N=100) |  | Non-DSPN (N=793) | DSPN (N=193) |  | Non-DSPN (N=817) | DSPN (N=392) |
| Male [n(%)] | 293(73.43%) | 75(75%) |  | 502(63.3%) | 140(72.54%) |  | 434(53.12%) | 282(71.94%)* |
| Age (y) | 34.63±7.61 | 34.05±8.03 |  | 52.98±4.12 | 53.42±3.97 |  | 67.46±6.38 | 69.26±6.88* |
| Duration of T2DM (y) | 1(0.08-4) | 4(0.3-8.25)* |  | 5(1-10) | 10(4-14)* |  | 10(3-15) | 12(7-20)* |
| Weight (kg) | 75.18±16.33 | 72.63±16.9 |  | 71.09±12.48 | 71±13.22 |  | 67.04±11.87 | 68.99±11.69* |
| Body mass index (kg/m^2^) | 26.21±4.68 | 25.05±4.65* |  | 25.57±3.62 | 25.06±3.87 |  | 25.33±3.61 | 25.14±3.61 |
| Systolic blood pressure (mmHg) | 126.49±17.03 | 125.8±16.56 |  | 129.44±15.6 | 132.31±18.96 |  | 135.97±17.7 | 135.47±18.73 |
| Diastolic blood pressure (mmHg) | 80.13±11.55 | 79.72±9.71 |  | 79.69±9.71 | 80.6±10.14 |  | 79.07±9.81 | 76.86±10.71* |
| HbA1c (%) | 9.42±2.38 | 10.05±2.63* |  | 8.61±2.06 | 9.81±2.37* |  | 8.6±2.07 | 9.32±2.1* |
| Total cholesterol (mmol/L) | 4.46(3.83-5.17) | 4.5(3.9-5.33) |  | 4.42(3.79-5.09) | 4.45(3.68-5.1) |  | 4.21(3.61-4.95) | 4.03(3.43-4.85)* |
| Triglycerides (mmol/L) | 1.77(1.15-2.78) | 1.67(1.02-2.42) |  | 1.54(1.09-2.3) | 1.47(0.98-2.3) |  | 1.37(1-1.99) | 1.22(0.88-1.79)* |
| LDL cholesterol (mmol/L) | 2.46(1.97-3.07) | 2.48(1.97-3.34) |  | 2.51(1.97-3.09) | 2.54(1.88-3.12) |  | 2.41(1.83-2.98) | 2.26(1.74-2.9)* |
| HDL cholesterol (mmol/L) | 0.98(0.82-1.19) | 1.05(0.87-1.27) |  | 1.06(0.88-1.25) | 1.07(0.86-1.32) |  | 1.07(0.9-1.31) | 1.07(0.89-1.34) |
| Serum creatinine (μmol/L) | 66(55-77) | 63(53-75.25) |  | 65(55-76) | 67(57-91)* |  | 69(56-82) | 76(62-93)* |
| Uric acid (μmol/L) | 330(259.5-403) | 336(272-433) |  | 309(250-370.25) | 314(241.5-392) |  | 304(252-368) | 315(258-380)* |
| eGFR (mL/min/1.73m^2^) | 105.68(86.17-118.87) | 110.71(88.47-121.57) |  | 95.71(79.3-105.47) | 91.06(64.61-105.05)* |  | 82.17(66.41-96.65) | 72.01(55.11-91.55)* |
| Calcium (mmol/L) | 2.28±0.11 | 2.25±0.15 |  | 2.26±0.12 | 2.23±0.16* |  | 2.26±0.12 | 2.24±0.13* |
| Phosphate (mmol/L) | 1.31±0.22 | 1.21±0.32* |  | 1.27±0.19 | 1.25±0.25 |  | 1.19±0.17 | 1.17±0.2 |
| Parathyroid hormone (ng/L) | 36.7(28.7-47) | 30.9(23.55-41.38)* |  | 36.9(29-47) | 32.2(24.4-41.7)* |  | 37.3(29.9-47.4) | 36(26.67-49.42) |
| 25(OH)D (nmol/L) | 37.75±17.75 | 31.65±15.7* |  | 42.06±17.15 | 36.15±18.08* |  | 41.62±19.84 | 37.36±20.82* |
| Vitamin D deficiency [n(%)] | 311(77.94%) | 88(88%)* |  | 568(71.63%) | 152(78.76%) |  | 571(69.89%) | 298(76.02%)* |

Abbrevation: DSPN: distal symmetric polyneuropathy; T2DM: type 2 diabetes, HbA1c: glycosylated hemoglobin, LDL: low-density lipoprotein, HDL: high-density lipoprotein, eGFR: estimated glomerular filtration rate.

* P＜0.05, vs. Non-DSPN.

Table S3. Odds ratios of vitamin D deficiency contributing to subclinical DSPN in different age groups.

|  | DSPN/  Non-DSPN | OR (95%CI) | P value | P for interaction |
| --- | --- | --- | --- | --- |
| Age (y) | - | - | - | <0.001 |
| Total (n=2694) | - | - | - | - |
| 25(OH)D ≥ 50 nmol/L | 147/559 | 1 (Ref) | - | - |
| 25(OH)D < 50 nmol/L | 538/1450 | 1.646(1.31-2.078) | <0.001 | - |
| Youth Group (n=499) | - | - | - | - |
| 25(OH)D ≥ 50 nmol/L | 12/88 | 1 (Ref) | - | - |
| 25(OH)D < 50 nmol/L | 88/311 | 2.427(1.201-5.308) | 0.018 | - |
| Middle-Age Group (n=986) - | | - | - | - |
| 25(OH)D ≥ 50 nmol/L | 41/225 | 1 (Ref) | - | - |
| 25(OH)D < 50 nmol/L | 152/568 | 1.96(1.288-3.039) | 0.002 | - |
| Elderly Group (n=1209) | - | - | - | - |
| 25(OH)D ≥ 50 nmol/L | 94/246 | 1 (Ref) | - | - |
| 25(OH)D < 50 nmol/L | 298/571 | 1.439(1.061-1.961) | 0.02 | - |

Multivariate adjusted: adjusted for sex, age, body mass index, blood pressure, glycosylated hemoglobin, T2DM duration, estimated glomerular filtration rate, calcium, phosphorus, parathyroid hormone, total cholesterol, triglyceride, low-density lipoprotein cholesterol and high-density lipoprotein cholesterol.
